# Airborne microalgal and cyanobacterial diversity and composition during rain events in the southern Baltic Sea region

**DOI:** 10.1038/s41598-022-06107-9

**Published:** 2022-02-07

**Authors:** Kinga A. Wiśniewska, Sylwia Śliwińska-Wilczewska, Anita U. Lewandowska

**Affiliations:** 1 Division of Marine Chemistry and Environmental Protection, Institute of Oceanography, University of Gdańsk, Av. M. Piłsudskiego 46, 81-378 Gdynia, Poland; 2 Division of Marine Ecosystems Functioning, Institute of Oceanography, University of Gdańsk, Al. M. Piłsudskiego 46, 81-378 Gdynia, Poland

**Keywords:** Ecology, Environmental sciences, Natural hazards

## Abstract

Airborne cyanobacteria and microalgae are commonly found in the atmosphere and may pose a serious human health risk. This study presents an innovative investigation of the washout efficiency of airborne cyanobacteria and microalgae in the Gulf of Gdańsk (southern Baltic Sea). For the first time, the number and type of cyanobacteria and microalgae were determined in rainwater samples and in air before and after rainfall events. The number of cyanobacteria and microalgae cells in the rainwater samples ranged, depending on, e.g., weather conditions, from 100 cells L^–1^ to 342.2 × 10^3^ cells L^–1^. Several harmful taxa, such as *Chlorococcum* sp., *Oocystis* sp., *Anabaena* sp., *Leptolyngbya* sp., *Nodularia* sp., *Pseudanabaena* sp., *Synechococcus* sp., *Synechocystis* sp., and *Gymnodinium* sp., were noted in our study. Washing out by rain is extremely relevant to human health and decreases the chance that people inhale these species and their toxic metabolic products. The greatest diversity of airborne microalgae and cyanobacteria was recorded in July 2019, despite this being the period with the lowest number of cells in rainwater samples. Research conducted in the southern Baltic Sea region confirmed the relationship between the occurrence of cyanobacteria and microalgae in the air and blooms in the sea. It is worth emphasizing that the number of microalgae and cyanobacteria cells decreased by up to 87% after a rainfall event relative to that before the rainfall event. The obtained results significantly increase the level of knowledge about cyanobacteria and microalgae present in the air. By demonstrating the washout efficiencies of cyanobacteria and microalgae, the results indicate the potential of individual taxa to be removed from the atmosphere with rainfall. The findings of this study are helpful for further research on airborne microorganisms and air quality.

## Introduction

The atmosphere contains diverse living microbes called bioaerosols. Among them, bacteria, viruses, fungi, pollen, microalgae, and cyanobacteria can be distinguished^1,2^. However, autotrophic organisms in the atmosphere are still poorly studied in comparison with heterotrophic organisms^3–8^. Cyanobacteria and microalgae present in the atmosphere are involved in cloud formation and influence the hydrological cycle and Earth’s climate^6,8,9^. Recent studies have demonstrated the negative health impacts of airborne cyanobacteria and microalgae, as well as the toxic compounds they produce^4,10,11^. The importance of these organisms in the atmosphere is described in detail elsewhere^3,4,8,9^. The present study focuses exclusively on the presence of cyanobacteria and microalgae in atmospheric aerosols and their wet deposition.

Depending on the prevailing weather conditions (e.g., wind speed, wind direction, temperature, air humidity)^12–14^, microorganisms, including cyanobacteria and microalgae, are emitted from water reservoirs or re-emitted from other surfaces to the atmosphere. The process is most effective during a period of high primary productivity in the oceans. According to Marshall and Chalmers^15^, air humidity is an important meteorological parameter in the cyanobacteria and microalgae emission process to the atmosphere. Marshall and Chalmers^15^ found that desiccation could increase the possibility of algae becoming airborne. Airborne microorganisms can subsequently be transported over long distances and/or incorporated into clouds before undergoing wet and/or dry deposition^6,8,9^. The first reports on this topic were reported during the 1970s, when it was suggested that heavy rainfall could lead to the intensive washout of airborne microalgae^16^. Sharma et al.^13^ noted that although rainfall removes airborne algae through the effects of rainout and washout, rainfall also releases algae through splash, tap, and puff mechanisms, thus emitting them into the air^17^.

Rain can efficiently remove airborne microbes. The microbial community in rain includes the microbes in the associated cloud as well as the air column below it. However, the diversity of microorganisms in rain is still poorly understood^18^. To date, scientists have detected cyanobacteria in clouds at an abundance ranging from ~ 1 to 50% of the total microbial community^19^. The size of the particles removed during atmospheric deposition determines their activity as cloud condensation nuclei, so determining the size of deposited particles is necessary to assess the effective removal from the atmosphere^20^.

Both wet and dry deposition are responsible for removing particles, including carbon and iron, from the atmosphere; however, wet deposition is more effective and can remove up to 80% of aerosols by mass^21–26^. Regarding the wet deposition of all atmospheric particles, both washout and rainout can remove cyanobacteria and microalgae from the atmosphere. Washout is a below-cloud process whereby aerosols are collected by falling hydrometeors. In contrast, rainout involves in-cloud scavenging, whereby particles act as cloud condensation nuclei in supersaturation conditions above the cloud^27^. Few studies have investigated the ability of cyanobacteria and microalgae to remain in the atmosphere and colonize new regions as a result of atmospheric deposition^28,29^. Airborne organisms may have an important impact on atmospheric processes and could also have an impact on ecosystems after their deposition. However, very few studies have investigated the presence of these organisms in clouds and rain.

In consideration of the aforementioned processes and dependencies, this study investigates the washout efficiency of airborne cyanobacteria and microalgae that may pose a human health risk. Many acute health problems related to bioaerosols have been identified, including asthma, allergic reactions, hay fever, skin inflammation, burning of the eyes, rhinitis, and respiratory irritation^2^. Accordingly, this study assesses whether cyanobacteria and microalgae are effectively removed from the atmosphere by determining the number and type of cyanobacteria and microalgae in rainwater samples and in air samples before and after the corresponding rainfall event.

## Results and discussion

This research focuses on the quantitative and qualitative analyses of cyanobacteria and microalgae present in rainfall during the summer phytoplankton bloom season of August–September 2019. In addition, a continuous episode of rainfall over several days was selected to demonstrate the washout process of microorganisms from the air with rain.

### Quantity of cyanobacteria and microalgae washed out with rain during the growing season

Currently, there is a growing number of scientific articles on cyanobacteria and microalgae in the atmosphere^8^. Unfortunately, there is a reference methodology for efficiently counting the microorganisms present in the air or in rainfall. A popular method for quantifying cyanobacteria and microalgae in the air is to show the number of taxa found in the collected samples after growth^6,31,42–46^. In this study, a total of 16 taxa of airborne cyanobacteria and microalgae were found in the samples. In the rainwater samples obtained during the summer of 2019, 11 taxa of cyanobacteria and microalgae were distinguished. The green algae in the rainwater samples included *Bracteacoccus* sp., *Oocystis* sp., *Coenochloris* sp., *Chlorella* sp., and *Chlorococcum* sp., while the cyanobacteria included *Leptolyngbya* sp., *Pseudanabaena* sp., *Synechococcus* sp., and *Synechocystis* sp. In addition, *Chrysochromulina* sp., which belongs to Haptophyta, was observed.

Other studies recorded the presence of several to several dozen taxa in the air^6,31,42–46^. Certainly, a number of factors, starting with atmospheric conditions and ending with physical and chemical parameters of the surrounding waters, influence the diversity of cyanobacteria and microalgae in the atmospheric air. Analyzing global trends, only cyanobacteria have been found in the atmosphere of every region of the world^31^. However, according to Dillon et al.^47^, cyanobacteria have been detected in clouds at variable abundances between ~ 1% and 50% of the total microbial community. Xu et al.^48^ found that cyanobacteria constituted only 1.1% of the total bacterial community in clouds. It needs to be highlighted that there is still a lack of research available to provide this type of information for rainfall samples.

**Figure 1:**
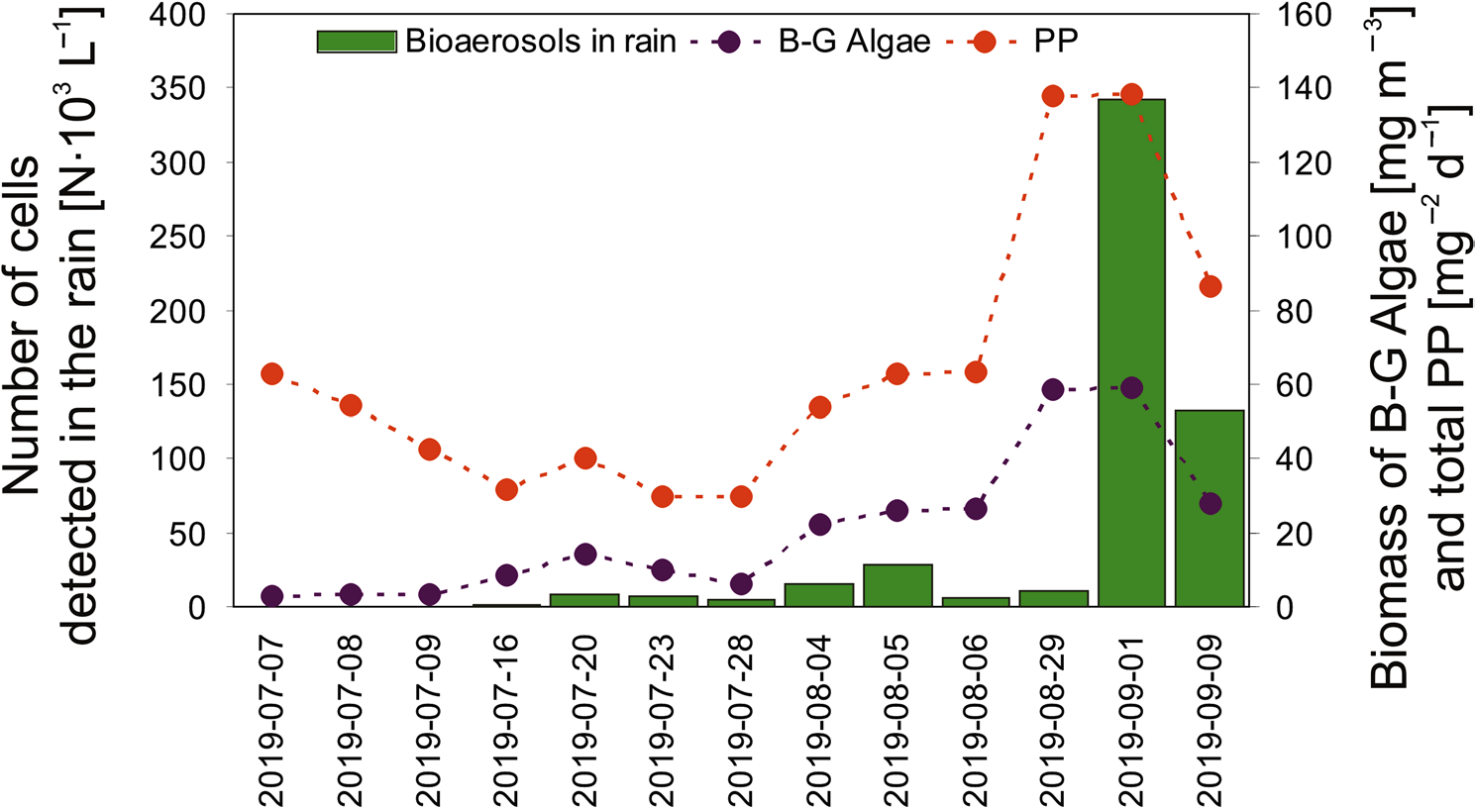
Number of cyanobacteria and microalgae cells present in the rainfall samples and the corresponding changes in their primary production (PP) and the biomass of cyanobacteria (B–G Algae) in the Gulf of Gdańsk (http://model.ocean.univ.gda.pl).

For the period from July to September 2019, the results showed that the number of cyanobacteria and microalgae cells present in rainfall varied over time (Fig. 1) and ranged between 100 cells L^–1^ and 342.2 × 10^3^ cells L^–1^. From July to the end of August, the cell number was relatively low, ranging from 100 cells L^–1^ to 28.6 × 10^3^ cells L^–1^. This variability was related to the change in the biomass of blue green algae in the Gulf of Gdańsk (Table S2; Fig. 1). Therefore, this research also shows the close relationship between the processes taking place in the Baltic Sea and the presence of cyanobacteria and microalgae in the atmosphere. As the biomass of cyanobacteria in the Baltic Sea increased, the number of cyanobacteria and microalgae cells in the rainfall samples also increased (***p < 0.001). This result may be representative of the dominant number of cyanobacteria cells in the rainfall over the Bay of Gdańsk. Based on the data from the hydrodynamic model (http://model.ocean.univ.gda.pl/) for the Bay of Gdańsk, intense increases in the biomasses of cyanobacteria and total phytoplankton in seawater were recorded at the beginning of September 2019 (Fig. 1). Moreover, when analyzing the meteorological conditions, the sudden increase in the biomass of cyanobacteria and microalgae in seawater could have been related to the relatively low wind speeds (mean of 1.3 m s^–1^ over a few days) and the highest air temperature (up to 31.2 °C on September 1) in the analyzed period (*p < 0.05 for air temperature). The influence of atmospheric pressure was also an important factor (***p < 0.001). It is known that the presence of cyanobacteria and microalgae in air, and subsequently in rainfall, is strongly related to the changes occurring in nearby seawater^8^. Moreover, the results of the present study revealed a high Spearman correlation between the number of cyanobacteria and microalgae cells in the rainwater samples and the NO_3_^–^ concentration of seawater (*p < 0.05) (Table S3). Therefore, these studies indirectly indicated that the processes leading to increased blooms in water bodies, with particular emphasis on blooms of toxic organisms, significantly affected the air quality in this region and could also influence the health of its citizens.

### Quality of cyanobacteria and microalgae washed out with rain during the growing season

Many studies have described the species composition of cyanobacteria and microalgae present in the atmosphere^2,4,6,12–14,16,42,43,46,49,50^. However, research on how these organisms are removed with precipitation from the atmosphere is still lacking. To the best of our knowledge, only Dillon et al.^47^ have reported on the number of microorganism taxa present in rain. Green algae of Trebouxiophyceae and cyanobacteria of Xenococcaceae were predominant in rainwater samples taken at the Opme meteorological station in France^47^. The authors classified the cyanobacteria in their rainwater samples as Phormidiaceae, Rivulariaceae, and Nostocaceae and the orders Pseudanabaenales and Synechococcales. Among the genre of green microalgae, *Chlorella* sp. was often observed by Dillon et al.^47^. Regardless of being in aerosols or rainwater, cyanobacteria and green algae have been found to be the dominant organisms^8,47^. Similar conclusions can be drawn from the results of the present study. In addition to cyanobacteria and green algae, *Chrysochromulina* sp. and *Gymnodinium* sp. were observed in the air aerosol samples during dry periods; however, they were not present in the subsequent rainfall samples. Differences in taxonomic composition between clouds and rainfall were reported by Dillon et al.^47^. Accordingly, we concluded that differences may exist between the taxonomic composition of aerosol and rain samples. Thus, in our opinion, there is a need for future in-depth research on the physics of microalgal and cyanobacterial particles removed from the air and clouds that would explain the exact reason why some organisms are washed out faster than others.

Among the microalgae and cyanobacteria present in the air, Genitsaris et al.^2^ distinguished those that have been shown to be harmful to human health once inhaled. These organisms can cause allergies, skin irritation, hay fever, rhinitis, and respiratory problems and may produce toxins. Several harmful taxa, such as *Chlorococcum* sp., *Oocystis* sp., *Anabaena* sp., *Leptolyngbya* sp., *Nodularia* sp., *Pseudanabaena* sp., *Synechococcus* sp., *Synechocystis* sp., and *Gymnodinium* sp., were observed in our study. However, on the one hand, presence in rainwater implies a successful purification process, but on the other hand, washout might result in the colonization of new regions. The origin of organisms in rainwater is related to their transport over marine waters, freshwater reservoirs, and terrestrial areas. According to Olenina^51^, most of the detected microalgae and cyanobacteria in rainwater and aerosol samples are typical of those in the Baltic Sea. Among them, we distinguished *Chlorella* sp., *Coenochloris* sp., *Oocystis* sp., *Anabaena* sp., *Leptolyngbya* sp., *Nodularia* sp., *Pseudanabaena* sp., *Synechococcus* sp., *Synechocystis* sp., *Gymnodinium* sp., and *Chrysochromulina* sp. According to Guiry and Guiry^52^, *Bracteacoccus* sp. and *Coccomyxa* sp. are freshwater and/or terrestrial taxa, while *Chlorococcum* sp. is a cosmopolitan taxon. *Coccomyxa* sp. has been previously found in air samples from the Baltic Sea region^29^. *Bracteacoccus* sp. and *Chlorococcum* sp. were isolated by Mikhailyuk et al.^53^ from biological soil crusts of maritime sand dunes of the Baltic Sea. In many respects, the Baltic is similar to an inland lake or an estuary and is unique because there are areas where freshwater, brackish water, and marine species are all present. Hence, the cyanobacteria and microalgae that we collected at our sampling station may have different salinity preferences. Wiśniewska et al.^29^ presented a detailed analysis of the salinity preferences of cyanobacteria and microalgae isolated from air samples.

### Cyanobacteria and microalgae washed out from the air: a case study

Although bacteria have been well studied, research in the area of airborne cyanobacterial and microalgal washout appears to be limited. The particular difficulty of this research is that it is impossible to plan a period of rainfall in advance. As there was no such period during the seasonal sampling in 2019, we performed additional measurements from August 27 to September 2, 2020, when there was almost daily intermittent rainfall. Aerosol samples were collected before and after each rainfall episode, and the qualitative and quantitative compositions of cyanobacteria and microalgae were determined in both sets of samples. In the rainwater samples, the observed cyanobacteria included *Anabaena* sp., *Synechococcus* sp., *Leptolyngbya* sp., and *Nodularia* sp., while the observed green algae included *Ankistrodesmus* sp., *Oocystis* sp., and *Stichococcus* sp. In the aerosol samples, the representative cyanobacteria were *Nodularia* sp. and *Synechococcus* sp., while the observed green algae included *Ankistrodesmus* sp., *Chlorella* sp., *Chlorococcum* sp., *Oocystis* sp., and *Stichococcus* sp. *Gymnodimium* sp. (Miozoa) and *Chrysochromulina* sp. (Haptophyta) were also observed in the aerosol samples. In the rain samples, 400–5000 cells L^–1^ were recorded during this period, whereas only 0.6–11.2 cells m^–3^ were measured in the aerosol samples (i.e., three orders of magnitude lower). The number of cyanobacteria and microalgae cells in the aerosols was comparable to that reported by Tormo et al.^54^ for samples collected in southwest Spain (0.18–3.85 cells m^–3^). The authors also found that the daily concentrations of microalgae and cyanobacteria in their air samples were positively correlated with temperature and wind speed and negatively correlated with rainfall and relative humidity.

**Figure 2:**
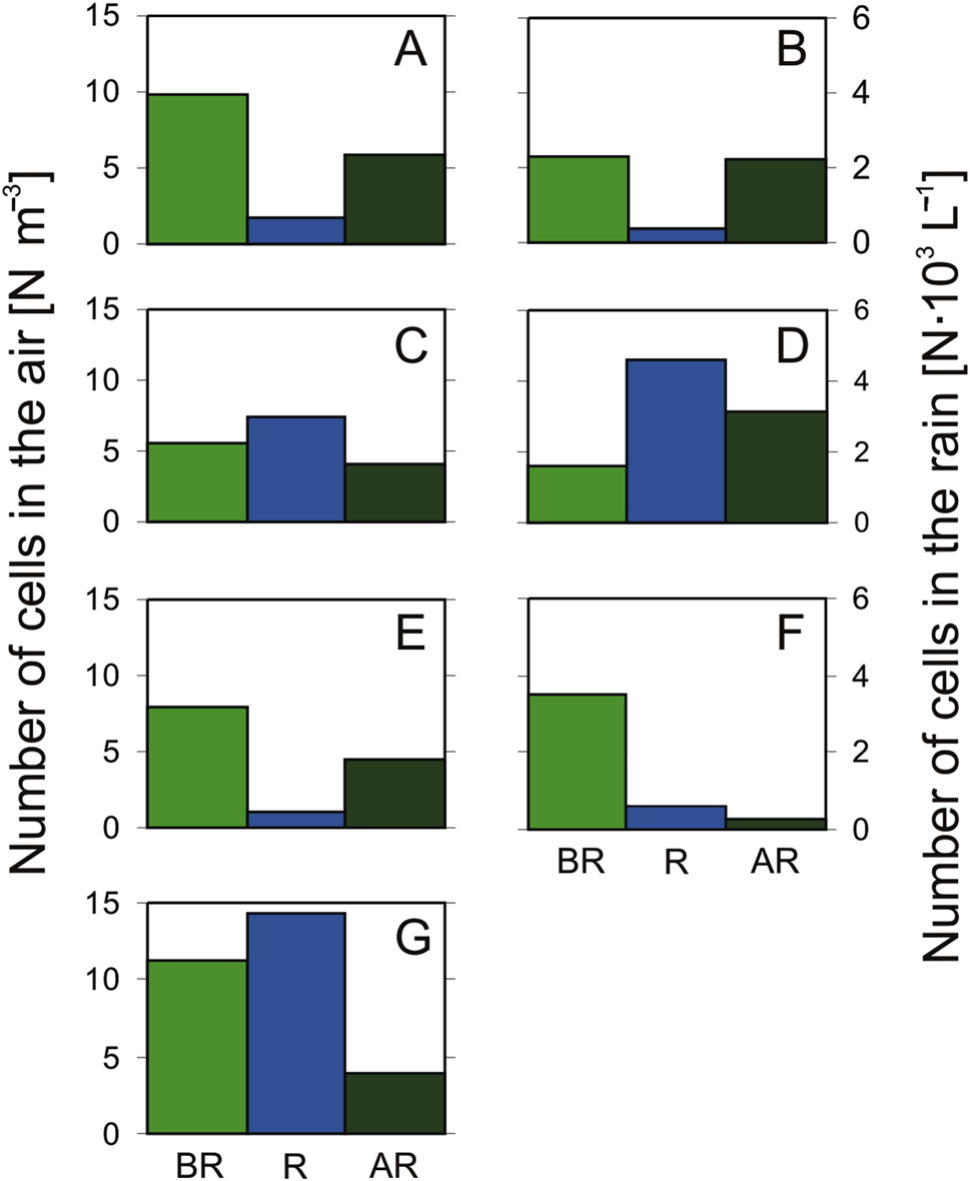
Number of microalgae and cyanobacteria cells in aerosol samples [cells m^–3^] before (BR) and after rainfall (AR) and in rain samples (R) [cells L^–1^] on the morning of August 25, 2020 (**A**), the afternoon of August 25, 2020 (**B**), on August 26, 2020 (**C**), at noon on August 27, 2020 (**D**), in the evening of August 27, 2020 (**E**), on August 28, 2020 (**F**), and from August 30 to September 1, 2020 (including 2 days of rainfall) (**G**).

The present research primarily aims to determine whether the presence of rainfall, as well as the number of microalgae and cyanobacteria cells recorded in it, influenced the number of cyanobacteria and microalgae cells in the air (Fig. 2). The results showed that the number of cyanobacteria and microalgae cells in the aerosol samples decreased by 21–87% after each rainfall event (relative to that prior to rainfall). The only exception was on August 27, when the number of microalgae cells increased significantly in the aerosol samples despite previous rainfall (Fig. 2D). On this day, sea air masses from the central Baltic Sea were transported over the measurement station (Fig. S1). The influx of air masses above the sea surface could have been associated with an increase in the microalgae and cyanobacteria taxa in the aerosol samples^6^. With the exception of this case, the largest decrease was 87% on August 29 (Fig. 2F), when the air mass trajectory after the period of rainfall changed from the north (carrying sea air masses) to the south (carrying inland air masses). A significant decrease (64%) in the number of microalgae and cyanobacteria cells in the aerosol samples was also observed after a period of rainfall lasting more than a day (Fig. 2G). This study is the first to discuss the effectiveness of the washing out of cyanobacteria and microalgae from the atmosphere with rain. It would be interesting to conduct similar types of research in other regions of the world, where the presence of cyanobacteria and microalgae, especially those that are harmful to human health, has also been demonstrated.

To date, the results obtained in this study can be compared only to the washing out of bacteria from the atmosphere. Research on washout conducted by Ouyang et al.^55^ showed that rainfall could remove up to 40% of bacteria from the atmosphere. However, we are not aware of any data in the literature regarding the washout efficiency of microalgae from the atmosphere. It should be noted that the number of microalgae and cyanobacteria cells present in rainwater does not necessarily mean that the cyanobacteria and microalgae that were in the air before the rainfall event were effectively removed. There was a case where a significant number of microalgae cells was found in a rainfall sample, but no decrease in the microalgae content of the aerosol sample was observed (Fig. 2D). This result may have been due to the continuous supply of cyanobacteria and microalgae from the sea, especially during strong phytoplankton blooms.

**Figure 3:**
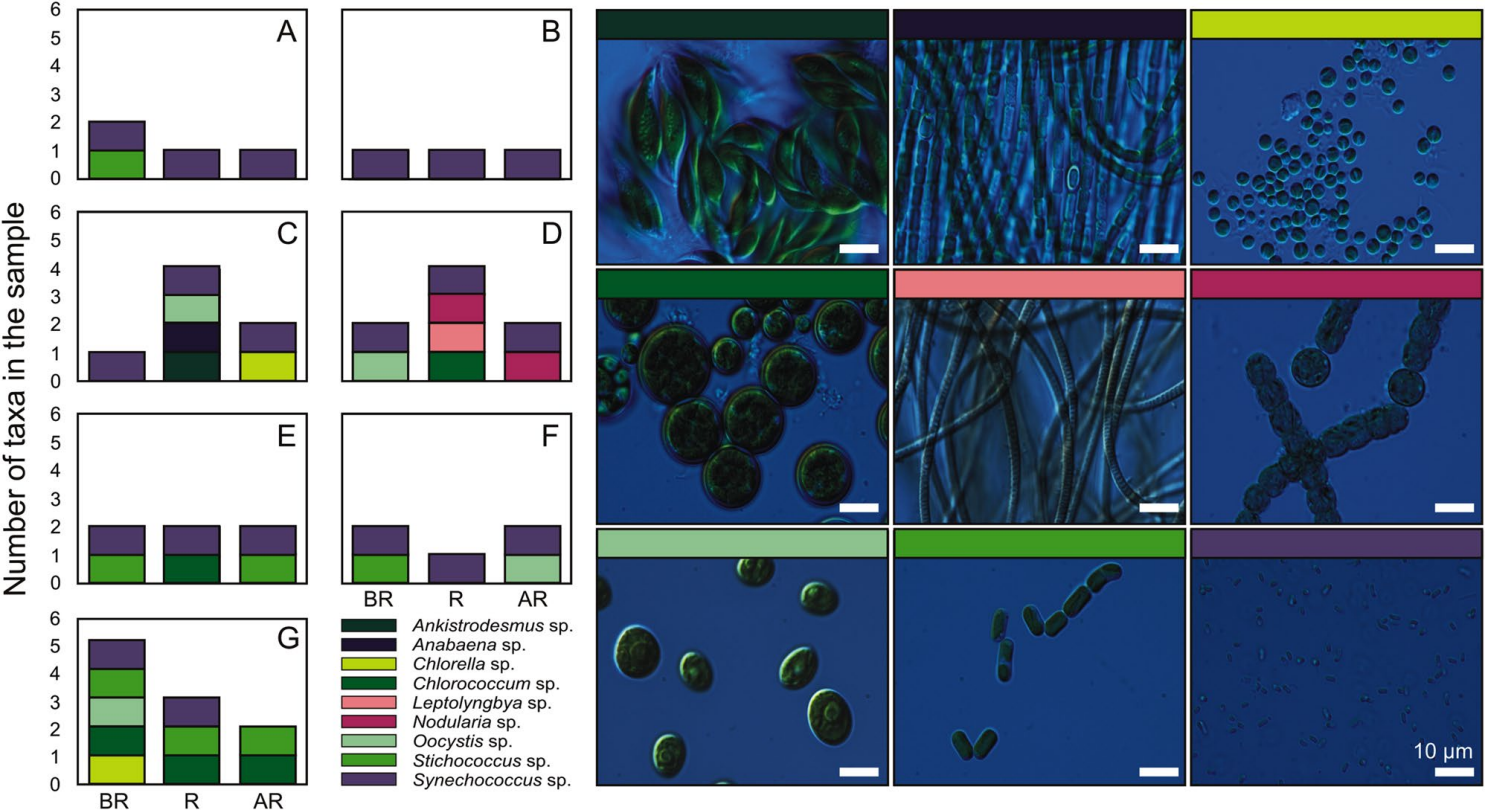
Number of microalgae and cyanobacteria cells in air samples before (BR) and after rainfall (AR) and in rainwater samples (R) on the morning of August 25, 2020 (**A**), the afternoon of August 25, 2020 (**B**), on August 26, 2020 (**C**), at noon on August 27, 2020 (**D**), in the evening on August 27, 2020 (**E**), on August 28, 2020 (**F**), from August 30 to September 1, 2020 (including 2 d of rainfall) (**G**) (left panel), and examples of microalgae and cyanobacteria collected from dry and wet deposition samples (right panel).

Dillon et al.^47^ found that cyanobacteria and microalgae were also present in clouds; thus, the microorganisms present in rainwater not only came from the aerosols present in the surrounding air but also could be washed out from clouds. Therefore, the taxonomic composition of rainwater and clouds^47^ may differ from that of aerosols. Most of the research on the washout of particles in air with rain has focused on bacteria. Joung et al.^56^ found that the amount of bacteria in the air after rainfall may significantly change. As a result of raindrops colliding with a substrate, bioaerosols can be re-emitted from the substrate to the air^56^. In the present study, an analysis of the taxonomic composition before and after periods of rainfall was also performed. Only on one occasion did the composition of the rainwater sample fully reflect the composition of the aerosol sample taken before the rainfall event, when *Synechococcus* sp. was observed in both samples (Fig. 3). There were no cases of a specific taxon being completely removed from the air by the rainfall event; however, for rainfall events lasting more than 24 h, *Synechococcus* sp. was completely washed out with the rain. This could have been related to the almost daily change in the direction of the air mass trajectory, whereby other taxa of microorganisms may have been supplied from slightly different source regions. Other studies have confirmed that the presence of new microalgae in a sample can be associated with a change in the air mass flowing over the measurement station^6,31^.

An interesting case was recorded after the rainfall event on August 27 (Fig. 3D), when the highest number of algae cells and the highest number of taxa were recorded in the rainwater sample. It is particularly interesting that *Nodularia* cf*. harveyana* was found in the rainwater sample because it was not observed in the aerosol samples before the rain, but it was found in the aerosol sample after the rainfall event. This result may suggest that, as in the case of bacteria, the re-emission of previously deposited particles could occur during intense rainfall^57^. Joung et al.^56^ found that when raindrops collided with soil, 0.01% of the total bacteria were emitted back into the air. Therefore, in the case of an increase in the amount of cyanobacteria and microalgae in the air, the re-emission of particles from the soil after rain should also be taken into consideration. However, this topic requires further detailed investigation. Additionally, after the rainfall event on August 27, two different species of *Nodularia* were recorded, as shown in Fig. 3D.

This research on washing out cyanobacteria and microalgae from the atmosphere by rain is pioneering and, therefore, definitely needs to be continued. We hope that our measurements will significantly influence the development of research on these organisms. In addition, it seems to be necessary to more extensively investigate the presence of cyanobacteria and microalgae in rain in different parts of the world. It would be advisable to learn more about the spatial variability and temporal variability of cyanobacteria and microalgae in rain. Our measurements were conducted for a relatively long time but only at one station. We would recommend further research on airborne cyanobacteria and microalgae regarding how they are washed out from the air at different kinds of research stations and at varying distances from the sea, both during the growing and the nonvegetative seasons. Information on airborne cyanobacteria, microalgae, and bacteria is summarized in Table S4.

## Conclusions

The results presented in the publication for the first time demonstrate the numbers of cyanobacteria and microalgae in rain. The number of cyanobacteria and microalgae cells in rainwater samples ranged from 100 cells L^–1^ to 342 × 10^3^ cells L^–1^. The taxonomic diversity as well as the numbers of airborne cyanobacteria and microalgae during changing meteorological conditions were thoroughly analyzed. The greatest diversity of airborne microalgae and cyanobacteria was recorded in July 2019, despite this being the period with the lowest number of cells in the rainwater samples. The highest number of cells for airborne microalgae and cyanobacteria corresponded to the highest concentration of phytoplankton in seawater, especially with respect to blue green algae. Thus, research conducted in the South Baltic Sea region confirmed the relationship between the occurrence of cyanobacteria and microalgae in the air and biochemical processes in the sea. Moreover, days of intensive rainfall favored the washing out of airborne microalgae and cyanobacteria. However, even a short dry period was sufficient to increase the number of cells again. Organisms were washed out of the atmosphere efficiently. The number of microalgae and cyanobacteria cells in aerosol samples decreased by up to 87% after a rainfall event with respect to that before the rainfall event. Rainfall had no significant effect on the taxonomic composition of cyanobacteria and microalgae, except when rainfall lasted more than 24 h. It is recommended that future research focus on developing methods to count cyanobacterial and microalgae particles in rain as well as in the atmosphere. In addition, it is particularly important to expand the research area on cyanobacteria and microalgae in rain. Increasing the emphasis to understanding the spatial variability and temporal variability of microalgae and cyanobacteria in rain and air, respectively, can also be crucial or fulfill existing gaps in the area of bioaerosol research.

## Methods

### Sampling location

Samples of airborne microalgae and cyanobacteria were collected at an observation station (20 m above sea level) on the roof of the Institute of Oceanography building in Gdynia (54° 31′ N, 18° 48′ E). The height of the building enables measurements to be taken from above the levels of neighboring tree canopies and buildings. The station is situated approximately 1 km from the Gulf of Gdansk coastal zone but is still in the city center and has been previously used for sampling bioaerosols, particulate matter, and rainfall^6,22,25,26^.

### Sample collection

In this study, two measurement campaigns were conducted during the period of highest primary production (PP) in the Baltic Sea. The first campaign was from May to September 2019, when rainwater samples were collected during periods of rainfall and air samples were collected. The second 1-week measuring campaign was from August 27 to September 2, when rainfall occurred almost every day, and aerosol samples were always collected before and after each rainfall event. In total, 20 rainwater samples and 11 samples of cyanobacteria and microalgae in aerosols were collected. The exposure time of the sample ranged from 30 min to 48 h depending on the rainfall duration. The collector was retracted as much as possible when it stopped raining.

The bulk rainfall collector consisted of a 1 dm^3^ polyethylene bottle with a small vent and a Teflon funnel with an area of 0.314 m^2^ for collecting rainfall. The bottle was tightly joined with the funnel and sealed by a Teflon ring. Before sampling, each bottle was treated with 1.0 M hydrochloric acid for 24 h and then rinsed three times with distilled and deionized water before being dried.

Prior to collecting the aerosol samples, a sterile mineral f/2 culture medium was prepared^30^ and calibrated using seawater with a salinity of 8 PSU. A combination of the methods used by Lewandowska et al.^6^ and Wiśniewska et al.^31^ was applied to collect bioaerosol samples. The samples in the liquid medium were placed in a biological impactor (Tisch Environmental, Inc.) consisting of six cascades that allowed particles of various diameters to be collected depending on the impactor cascade (1) > 7 μm; (2) 4.7–7 μm; (3) 3.3–4.7 μm; (4) 2.1–3.3 μm; (5) 1.1–2.1 μm; (6) < 1.1 μm). The impactor containing Petri dishes with liquid f/2 medium (6 mL) was exposed for between 30 min and 6 h depending on the rainfall duration. Samples were taken during the day and night. The sampler air flow was 28.3 L min^–1^.

### Sample preservation until analysis

To cultivate the microalgae and cyanobacteria present in the rainwater samples, the components of the f/2 medium were added to 20 mL of rainwater in at least one repetition depending on the sample volume. The rainwater and bioaerosol samples were grown for 30 d under a constant temperature of 20 °C on a 16:8 h light:dark cycle at 10 μmol photons m^–2^ s^–1^. The intensity of photosynthetically active radiation (PAR) was measured using a quantum meter (LI-189, LI-COR Inc., Nebraska, USA) with a cosine collector.

### Identification of taxonomic composition and number of identified taxa in the collected material

The taxonomic composition and number of identified taxa were determined using a light microscope (Nikon Eclipse 80i, Nikon, Tokyo, Japan) equipped with a camera (Nikon DSU2, Plan Apo VC 100 objective; magnification of × 1000). In addition, to verify the studied material, an epifluorescence microscope (Nikon Eclipse 80i, Nikon, Tokyo, Japan) with UV-2A, B-2A, and G-2A block filters was used. The latter proves the chlorophyll *a* content in the identified taxa and thus the ability to conduct photosynthesis processes. This fluorescence is also widely used in plant physiology as an indicator of the condition of chloroplasts and algal cells^32,33^.

The analyzed material was collected from Petri dishes and later transferred (in triplicate) into 5-mL plastic tubes. It was then checked under a light and epifluorescence microscope. Phytoplankton organisms were identified at the species level or, if this was impossible, at the genus level. Taxa were identified using keys and relevant literature^34–37^. A 20-mL sample was used to determine the number of microorganism cells in each rainwater sample. The number of cyanobacterial and microalgal cells (N) in bioaerosols was counted by a flow cytometer (BD Accuri™ C6 Plus; BD Biosciences, San Jose, California, USA). Detectors FL1, FL2, and FL3 read the fluorescence emissions excited by the blue laser (480 nm), while detector FL4 read the emissions excited by the red laser (640 nm)^38^. In the bioaerosol samples, the populations of cyanobacteria and microalgae were examined using flow cytometry and an epifluorescence microscope. In the case of filamentous cyanobacteria, the individual cells in the filaments were counted separately according to the method proposed by Śliwińska-Wilczewska et al.^39^.

### Meteorological data and other parameters

Meteorological data supplied by ARMAAG (https://armaag.gda.pl/) were used to supplement the results (Table 1). Additionally, 48 h backward trajectories were determined using the Hybrid Single-Particle Lagrangian Integrated Trajectory (HYSPLIT)^40,41^ model to approximate the air mass source (Fig. S1). The results were also supplemented with chemical analysis data (e.g., NO_3_^2–^ and PO_4_^3–^) of the rainwater and seawater samples^39^ (Table S1). The ecohydrodynamic model http://model.ocean.univ.gda.pl) was used to estimate data for the blue green algae biomass and total primary production in the Baltic Sea (Table S2).

**Table 1:** The average of the meteorological parameters during the sampling days and the summed amount of precipitation.

Date	Air temp [°C]	Relative humidity [%]	Wind velocity [m s^–1^]	Atmospheric pressure [hPa]	Amount of precipitation [mm]
07.07.2019	16.52	64.23	1.67	1004.15	5.97
08.07.2019	16.15	80.13	1.92	1007.20	21.85
09.07.2019	16.31	79.99	1.55	1008.65	3.01
16.07.2019	22.70	67.21	1.12	1010.38	6.33
20.07.2019	18.78	68.79	1.15	1010.04	2.18
23.07.2019	20.55	77.92	0.89	1013.14	0.13
28.07.2019	22.10	75.20	2.11	1007.99	3.44
04.08.2019	19.17	66.38	0.52	1012.05	1.32
05.08.2019	19.10	77.81	1.30	1013.20	7.80
07.08.2019	20.72	70.41	1.93	1012.69	14.91
29.08.2019	22.39	68.45	1.27	1012.81	4.58
01.09.2019	24.67	56.85	1.33	1016.75	6.70
09.09.2019	22.77	81.44	1.86	1018.85	5.71
25.08.2020	16.31	72.39	1.72	1009.14	5.29
26.08.2020	16.70	73.15	1.68	1001.29	4.66
27.08.2020	17.27	75.15	2.28	1003.25	13.35
28.08.2020	16.60	67.78	1.48	1007.66	2.37
29.08.2020	17.84	75.15	2.28	1003.25	–
30.08.2020	17.89	73.20	0.95	1007.98	–
31.08.2020	17.46	72.99	2.14	1010.48	3.16
01.09.2020	15.36	63.22	2.05	1016.49	5.91
02.09.2020	16.13	78.17	2.52	1012.76	–
03.09.2020	15.34	78.49	1.35	1013.20	–

– means no rain event.

### Statistical analysis

Spearman correlation coefficients were calculated between the number of microalgae and cyanobacteria cells in the rainwater samples (cells L^–1^) and the daily rainfall amount (mm), mean temperature (**°**C), relative humidity (%), atmospheric pressure (hPa), wind speed (m s^–1^), NO_3_^–^ concentration in seawater (mg m^–3^), PO_4_^3–^ concentration in seawater (mg m^–3^), blue green algae biomass in the Baltic Sea (mg m^–3^), and primary production (mg m^–3^) in the Baltic Sea (Table S3). Asterisks are used to indicate a significant difference compared with the control as follows: *p < 0.05; **p < 0.01; ***p < 0.001.

## Supplementary Information

Supplementary Information.

## Data Availability

All data generated or analyzed during this study are included in this article (and its Supplementary Information files).
