# Supplementary material for: Airborne microalgal and cyanobacterial diversity and composition during rain events in the southern Baltic Sea region
Source: Sci Rep. 2022 Feb 7;12:2029. doi: 10.1038/s41598-022-06107-9 (PMC8821709; doi:10.1038/s41598-022-06107-9)
Supplement: Supplementary file 1 — Supplementary Information. [file 41598_2022_6107_MOESM1_ESM.doc]

**Supplementary material**

| **Sample ID** | **Date of sampling** | **NO_3_^2–^ [mg L^–1^]** | **PO_4_^3–^ [mg L^–1^]** |
| --- | --- | --- | --- |
| R0119 | *05/07/19* | - | - |
| R0219 | *06/07/19* | - | - |
| R0319 | *07/07/19* | 0.5 | 0.01 |
| R0419 | *08/07/19* | 2.4 | 0.05 |
| R0519 | *09/07/19* | 3.4 | 0.02 |
| R0619 | *16/07/19* | 0.8 | 1.23 |
| R0719 | *20/07/19* | 3.2 | 5.64 |
| R0819 | *23/07/19* | 0.8 | 0.90 |
| R0919 | *28/07/19* | 0.6 | 0.03 |
| R1019 | *04/08/19* | - | 0.82 |
| R1119 | *05/08/19* | 0.8 | 0.05 |
| R1219 | *06/08/19* | 1.2 | 0.09 |
| R1319 | *09/08/19* | 0.9 | 0.08 |
| R1419 | *01/09/19* | 1.6 | 1.65 |
| R1519 | *09/09/19* | 0.7 | 0.32 |
| R0120 | *25/08/20* | - | - |
| R0220 | *25/08/20* | - | - |
| R0320 | *25/08/20* | 0.8 | 0.14 |
| R0420 | *26/08/20* | - | - |
| R0520 | *27/08/20* | 0.6 | 0.36 |
| R0620 | *28/08/20* | 0.9 | 1.18 |
| R0720 | *31/08/20* | 1.1 | 0.54 |
| R0820 | *01/09/20* | 0.5 | 0.05 |

**Table S1.** Nutrients measured in the rain sample (http://model.ocean.univ.gda.pl).

| **Date of sampling** | **NO_3_^2–^**  **[mg m^–3^]** | **PO_4_^3–^**  **[mg m^–3^]** | **B-G Algae**  **[mg m^–3^]** | **PP**  **[mg m^–2^ d^–1^]** |
| --- | --- | --- | --- | --- |
| *05/07/19* | 11.6 | 14.3 | 3 | 15.7 |
| *06/07/19* | 11.2 | 14.2 | 3 | 12.1 |
| *07/07/19* | 12.4 | 14.3 | 3 | 7.76 |
| *08/07/19* | 13.6 | 14.5 | 3.2 | 5.96 |
| *09/07/19* | 16.1 | 14.4 | 3.1 | 2.85 |
| *16/07/19* | 24.6 | 14.1 | 8.3 | 4.38 |
| *20/07/19* | 45.6 | 17.6 | 14.1 | 5.72 |
| *23/07/19* | 81.4 | 25.2 | 9.7 | 2.35 |
| *28/07/19* | 287 | 58.7 | 6.3 | 4.96 |
| *04/08/19* | 185 | 41.7 | 22 | 5.84 |
| *05/08/19* | 196 | 42.4 | 26.1 | 8.23 |
| *07/08/19* | 191 | 42.3 | 26.4 | 6.37 |
| *29/08/19* | 208 | 40.8 | 57.1 | 17.2 |
| *01/09/19* | 181 | 39.8 | 59.2 | 23.1 |
| *09/09/19* | 159 | 36.4 | 28 | 19.5 |
| *25/08/20* | 163 | 48.3 | 6.7 | 6.15 |
| *26/08/20* | 147 | 48.3 | 6.6 | 8.69 |
| *27/08/20* | 153 | 45.9 | 7.5 | 8.22 |
| *28/08/20* | 152 | 44.5 | 10.5 | 6.7 |
| *29/08/20* | 160 | 45.3 | 6.3 | 5 |
| *30/08/20* | 161 | 46.7 | 7 | 7.81 |
| *31/08/20* | 164 | 44.7 | 8.7 | 5.27 |
| *01/09/20* | 163 | 45 | 11.5 | 7.39 |
| *02/09/20* | 179 | 45.7 | 10.2 | 4.44 |

**Table S2.** The nutrient composition in the Gulf of Gdansk (Baltic Sea), phytoplankton biomass – blue green algae biomass and the primary production (http://model.ocean.univ.gda.pl).

|  | **Rainfall** | **T_mean_** | **Rh** | **hPa** | **Ws** | **NO_3_^2–^** | **PO_4_^3–^** | **BG biomass** | **PP** |
| --- | --- | --- | --- | --- | --- | --- | --- | --- | --- |
| Microalgae and cyanobacteria in rain | -0.098 | 0.604 | -0.105 | 0.78 | -0.302 | 0.588 | 0.549 | 0.890 | 0.165 |
| *p* value | >0.05 | *<0.05 | >0.05 | ***<0.001 | >0.05 | *<0.05 | >0.05 | ***<0.001 | >0.05 |

**Table S3.** Spearman rank correlation coefficients between number of microalgae and cyanobacteria in the rain (cells L^–1^) and: daily records for rainfall [mm], mean temperature [°C], relative humidity [%], atmospheric pressure [hPa], wind speed [m s^–1^], NO_3_^2–^ [mg m^–3^] and PO_4_^3–^ [mg m^–3^] concentration in sea water, blue green algae biomass [mg m^–3^] and primary production [mg m^–2^ d^–1^] in the Baltic Sea.

| **Environment** | **Type of bioaerosol** | **Type of research** | **References** |
| --- | --- | --- | --- |
| Aerosols | Cyanobacteria | Quality | El-Gamal [44] |
| Aerosols | Cyanobacteria, microalgae | Quantity and quality | Genitsaris et al. [2] |
| Aerosols | Cyanobacteria, microalgae | Quality | Lee and Eggleston [43] |
| Aerosols | Cyanobacteria, microalgae | Quality | Lewandowska et al. [6] |
| Aerosols | Cyanobacteria, microalgae | Quantity and quality | Marshall et al. [15] |
| Aerosols | Cyanobacteria | Quantity | Murby and Haney [10] |
| Aerosols | Cyanobacteria, microalgae | Quality | Ng et al. [46] |
| Aerosols | Bacteria, cyanobacteria | Quality and quantity | Jang et al. [57] |
| Aerosols | Cyanobacteria, microalgae | Quantity and quality | Rosas et al. [12] |
| Aerosols | Cyanobacteria, microalgae | Quality and quantity | Sharma and Singh [45] |
| Aerosols | Cyanobacteria, microalgae | Quantity and quality | Sharma et al. [13] |
| Aerosols | Cyanobacteria, microalgae | Quantity and quality | Singh et al. [14] |
| Aerosols | Cyanobacteria, microalgae | Quality and quantity | Tormo et al. [54] |
| Aerosols | Cyanobacteria, microalgae | Quality | Wiśniewska et al. [31] |
| Aerosols, rainfall | Cyanobacteria, microalgae | Quantity and quality | Ouyang et al. [55] |
| Aerosols, soil | Cyanobacteria, microalgae | Quantity and quality | Carson et al. [16] |
| Aerosols, soil, buildings | Cyanobacteria, microalgae | Quality and quantity | Chu et al. [50] |
| Aerosols, soil, water | Cyanobacteria, microalgae | Quality and quantity | Sharma et al. [49] |
| Cloud water | Bacteria, Cyanobacteria | Quantity and quality | Kourtev et al. [19] |
| Cloud water | Bacteria, Cyanobacteria | Quantity and quality | Xu et al. [48] |
| Rainfall | Bacteria | Quantity and quality | Joung et al. [56] |
| Rainfall, clouds | Cyanobacteria, microalgae | Quantity and quality | Dillon et al. [47] |
| Aerosols, rainfall | Cyanobacteria, microalgae | Quantity and quality | Current study |

**Table S4.** Comparing the information on the type of bioaerosols, the location of airborne cyanobacteria and microalgae as well as the type of research performed.


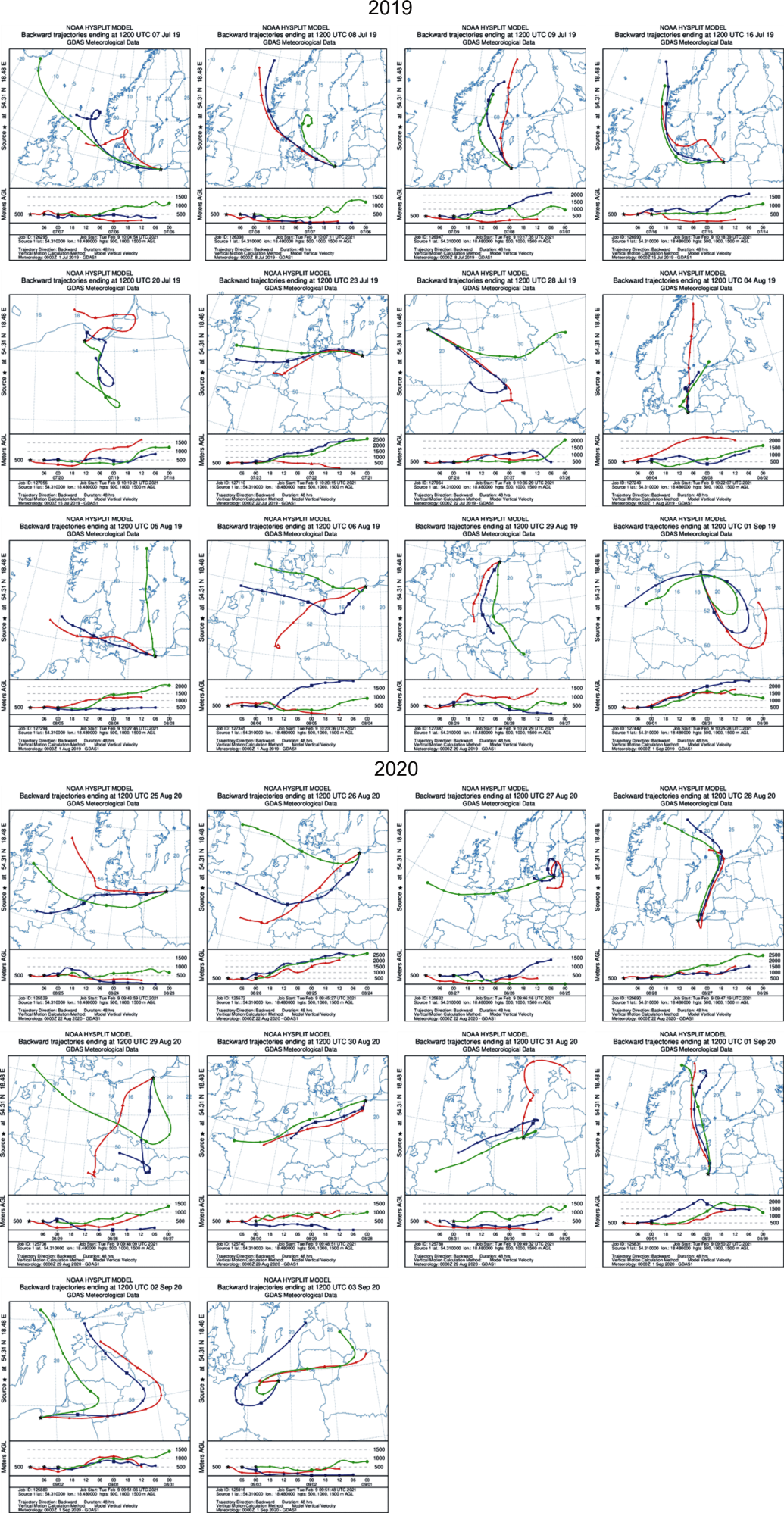


**Figure S1.** Representative 48 h backward trajectories of air masses during sampling period (HYSPLIT https://www.ready.noaa.gov).
